# Influence of conductive carbon and MnCo_2_O_4_ on morphological and electrical properties of hydrogels for electrochemical energy conversion

**DOI:** 10.3762/bjnano.15.6

**Published:** 2024-01-11

**Authors:** Sylwia Pawłowska, Karolina Cysewska, Yasamin Ziai, Jakub Karczewski, Piotr Jasiński, Sebastian Molin

**Affiliations:** 1 Faculty of Electronics, Telecommunications and Informatics, and Advanced Materials Center, Gdańsk University of Technology, G. Narutowicza St. 11/12, 80-233 Gdańsk, Poland; 2 Institute of Fundamental Technological Research, Polish Academy of Sciences, Pawińskiego St. 5B, 02-106 Warsaw, Poland; 3 Faculty of Applied Physics and Mathematics, and Advanced Materials Center, Gdańsk University of Technology, G. Narutowicza St. 11/12, 80-233 Gdańsk, Poland

**Keywords:** electrical properties, energy, hydrogel, hydrogen, oxygen evolution reaction, polymer composites

## Abstract

In this work, a strategy for one-stage synthesis of polymer composites based on PNIPAAm hydrogel was presented. Both conductive particles in the form of conductive carbon black (cCB) and MnCo_2_O_4_ (MCO) spinel particles were suspended in the three-dimensional structure of the hydrogel. The MCO particles in the resulting hydrogel composite acted as an electrocatalyst in the oxygen evolution reaction. Morphological studies confirmed that the added particles were incorporated and, in the case of a higher concentration of cCB particles, also bound to the surface of the structure of the hydrogel matrix. The produced composite materials were tested in terms of their electrical properties, showing that an increase in the concentration of conductive particles in the hydrogel structure translates into a lowering of the impedance modulus and an increase in the double-layer capacitance of the electrode. This, in turn, resulted in a higher catalytic activity of the electrode in the oxygen evolution reaction. The use of a hydrogel as a matrix to suspend the catalyst particles, and thus increase their availability through the electrolyte, seems to be an interesting and promising application approach.

## Introduction

Hydrogels are defined as a group of polymeric materials with an insoluble hydrophilic structure which gives them the ability to absorb and hold large amounts of water (up to over 99 wt %) in their three-dimensional network. The phenomenon in which hydrogels swell in water while not dissolving in it is due to hydrophilic functional groups attached to the polymer backbone and cross-links between the network chains. High water content makes hydrogel materials similar in terms of microstructure and flexibility to living tissues. The amount of water absorbed depends on factors such as the structure of the hydrogel, the composition of the precursor hydrogel solution, the cross-link density, and the technique of its synthesis. The simple reaction of one or more monomers is used for the production of this kind of material [1–3]. The methods of synthesising hydrogels are divided into two basic groups, including physical and chemical cross-linking. Physical cross-linking methods, which are mainly related to the synthesis of natural hydrogels, include changes in intermolecular interactions (e.g., hydrophobic interactions, ionic cross-linking, and hydrogen-bonded gels). Chemically synthesised hydrogels are produced by covalent cross-linking pathways such as radical polymerisation, radiation cross-linking, grafting, thermogelation, enzymatic reactions, and click chemistry [4–5].

Hydrogel materials have quite a long history and a wide range of applications, especially in biology, medicine, tissue engineering, and pharmacy. The multitude of application areas is related to their exceptional properties: biocompatibility, biodegradability, nontoxicity, good permeability for substances dissolved in water (e.g., oxygen and metabolites), flexibility with high mechanical strength, chemical and thermal resistance, high rate of reversible fluid absorption, and low interfacial tension with water [6–7]. A very desirable property of hydrogels is the ability to incorporate or suspend various particles in their structure, such as dyes, drugs, metal nanoparticles, metal oxide nanoparticles, carbon nanotubes, or biomolecules. This is a very important advantage that opens ways of designing composite hydrogels with various properties and applications such as biomedical [8–10], biosensors [11–13], wearable electronics [14–16], and environmental [17–18]. In recent years, scientists have been very interested in the use of hydrogels in electrocatalytic water splitting to produce hydrogen from renewable energy sources. These studies assume the use of empty spaces, thus ensuring efficient mass transport, as well as increasing electrochemically active surfaces. The great advantage of the 3D hydrogel structure is the increase of the catalytic surface area thanks to the possibility of conducting the electrochemical reaction deeper into the structure of the hydrogel. Generation of free space in the form of pores and network structure within the morphology also facilitates the penetration by the electrolyte, the diffusion of ions to electroactive sites, and the rapid release of the reaction, thus promoting the kinetics of the reaction and achieving higher efficiency of the catalyst built into the 3D structure [19]. The hydrogel matrix porous structure is capable of swelling and thus accommodating large amounts of ionic liquids. Moreover, the swollen hydrogel structure provides constant access of electrolyte molecules/ions to the catalyst particles, increasing the speed and efficiency of the electrochemical reaction [20]. The swelling of the polymer hydrogel in an aqueous environment makes this unlikely choice of material very important, and the optimal combination of polymer and catalyst can provide a robust and efficient electrode system in which the aqueous electrolyte is absorbed in the polymer matrix. This structure is designed to facilitate and increase mass transport; therefore, it is vital to achieve the desired three-dimensional structure of the material. In addition, the free space in the form of pores and the network structure within the polymer facilitate the penetration by the electrolyte, the diffusion of ions to electroactive sites, and the rapid release of the reaction, thus promoting the kinetics of the reaction and achieving higher efficiency of the catalyst built into the hydrogel structure [19,21]. The condition which must be met for hydrogels to be used in energy conversion systems is their appropriate electrical conductivity [22]. Suspension of conductive fillers in the hydrogel structure, such as metallic particles (gold nanoparticles, silver nanoparticles) [23–25], carbon-based materials (GO graphene oxide, CNT carbon nanotubes) [26–28], and conductive polymers (polyaniline, polypyrrole, and poly(ethylenedioxythiophene) [29–31], allows for the formation of conductive hydrogels. Conducting hydrogels possess properties of both conductive polymers and hydrogels, which makes them attractive functional materials for many applications in several fields of science and technology, such as environmental engineering [32], renewable energy [22,33–35], electronics [36–38], medical devices [39–41], and drug delivery systems [42–45]. They combine the properties of a hydrophilic matrix with conductive properties obtained thanks to the use of an appropriate conductive component at the stage of hydrogel synthesis. Hydrogel matrices are an excellent medium for depositing metallic nanocatalysts, especially in reactions requiring aqueous media such as electrochemical processes. The dispersion of the catalyst particles in the hydrogel helps to avoid or significantly reduce the formation of aggregates, increase the active surface area of the catalyst, and thus affects its efficiency [20]. In standard systems used in energy conversion processes, where the catalytic layer is either a thin layer consisting of catalyst particles, conductive fillers, and binder agents (e.g., Nafion), or three-dimensional systems created by a hydrogel matrix, the presence of conductive fillers (e.g., conductive carbon black) is crucial. This addition allows to obtain a conductive hydrogel and thus increase the number of conduction paths. The increase in the conductive properties of the hydrogel contributes to the improvement of the electronic conductivity of weakly conductive electrocatalysts, such as metal oxides, ultimately affecting their catalytic efficiency and thus reducing the overpotential of the oxygen evolution reaction (OER) process [46].

In this work, we suspended particles of the MnCo_2_O_4_ electrocatalyst and conductive carbon in a PNIPAAm hydrogel precursor solution, which was then subjected to polymerisation. As a result, a hydrogel composite was created in a single-stage synthesis process. In addition, the applied methodology allowed us to avoid the need to use elevated temperatures at the synthesis stage, which is an essential step in most of the conventional synthesis paths of conductive hydrogels described in the literature [23–24,26–28]. These two features, the one-stage process, and the low-temperature needed, are used for synthesising the polymer composite with significant electrical conductivity resulting from the presence of conductive carbon particles in a process that is much faster and easier to perform. It is also inexpensive as it does not require electricity consumption in long mixing and/or heating processes. The produced hydrogel structures were examined in terms of their morphology, electrical properties, and catalytic layers in the OER process.

## Results and Discussion

### Characterisation of hydrogel-based polymer composites with dispersed catalytic and conductive particles

**Figure 1:**
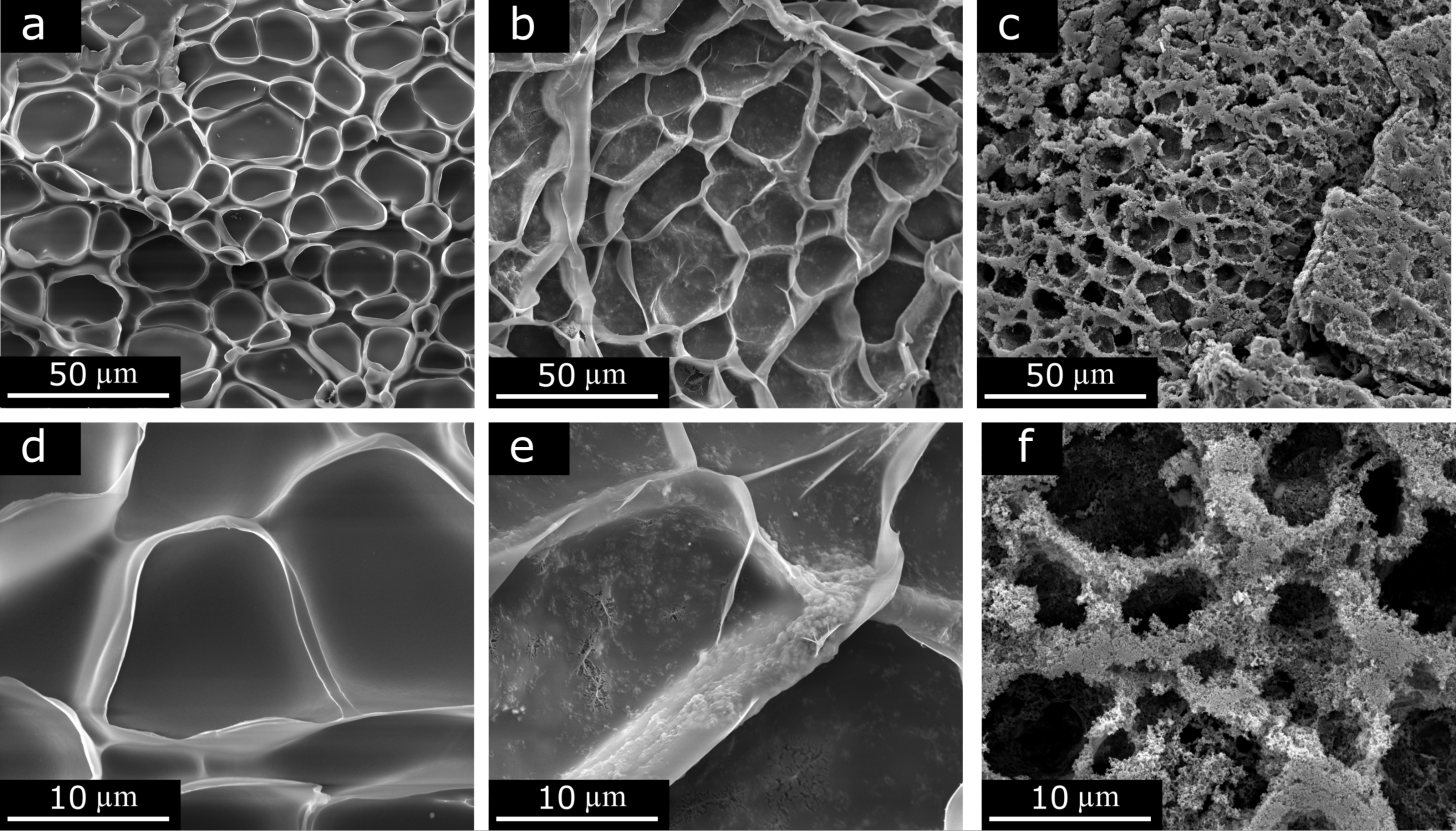
SEM micrographs of freeze-dried nanostructures of pure (a, d) and composite hydrogel samples: Hgel-MCO-cCB 1:3 (b, e) and Hgel-MCO-cCB 1:6 (c, f).

Scanning electron microscopy (SEM) analysis of hydrogel samples subjected to lyophilisation was performed to determine the presence of catalyst particles and conductive carbon on the surface of the porous skeleton created by the PNIPAAm polymer. Figure 1a and Figure 1d show a porous structure, with pure hydrogel as the skeleton. Higher magnification of the polymer surface for the sample Hgel-MCO-cCB 1:3 showed the presence of MCO and cCB particles in the hydrogel scaffold, confirming their entrapment in the polymer matrix (Figure 1b and Figure 1e). A similar composite structure with gold nanorods suspended in the PNIPAAm matrix was observed by Nakielski et al. [47]. Cai et al. found some silver nanoparticles only slightly present in the skeleton of a PVA@DEL composite hydrogel [23]. Most of the Ag nanoparticles were observed in the wall of the hydrogel network. In our case, for the Hgel-MCO-cCB 1:3 sample, when the volume percentage of cCB was 17.1% (Table 3), the particles of conductive carbon and catalyst were incorporated into the structure of the hydrogel matrix. Increasing the volumetric contribution of cCB particles above this value (e.g., for the Hgel-MCO-cCB 1:6 sample) resulted in partial release of these particles onto the surface of the polymer matrix (Figure 1c, Figure 1f). However, the samples were not homogeneous in terms of the distribution of composite particles in the hydrogel structure. Even at high concentrations of conductive particles, there were regions where the hydrogel skeleton was covered with cCB particles, while inside the backbone, very few of them were observed. Therefore, it can be concluded that there is a certain limit to the concentration of particles added to the hydrogel, which after the polymerisation process are trapped in the hydrogel skeleton. Above this limit, excess particles are still bound to the polymer scaffold, coating its surface. The characteristic porous structure is still formed but has a much more granular structure. The presence of pores is very important in the case of the application of hydrogel composite in OER. As aforementioned, the porosity has a beneficial effect on the diffusion of electrolytes in an electrochemical process. It provides a greater effective surface area and facilitates the transport of electrons and ions from the electrolyte to the electrocatalyst surface. As the morphological analysis showed, with the increase in the concentration of conductive carbon particles, the size of the pores and their number decreases (data presented in Figure 1 and Supporting Information File 1, Figure S1, and Figure S2). Further research should focus, among other topics, on finding the right proportions between the components of hydrogel composites to achieve a balance between the influence of porosity and conductive properties of these composites on the electrochemical activity of catalyst particles in OER.

**Figure 2:**
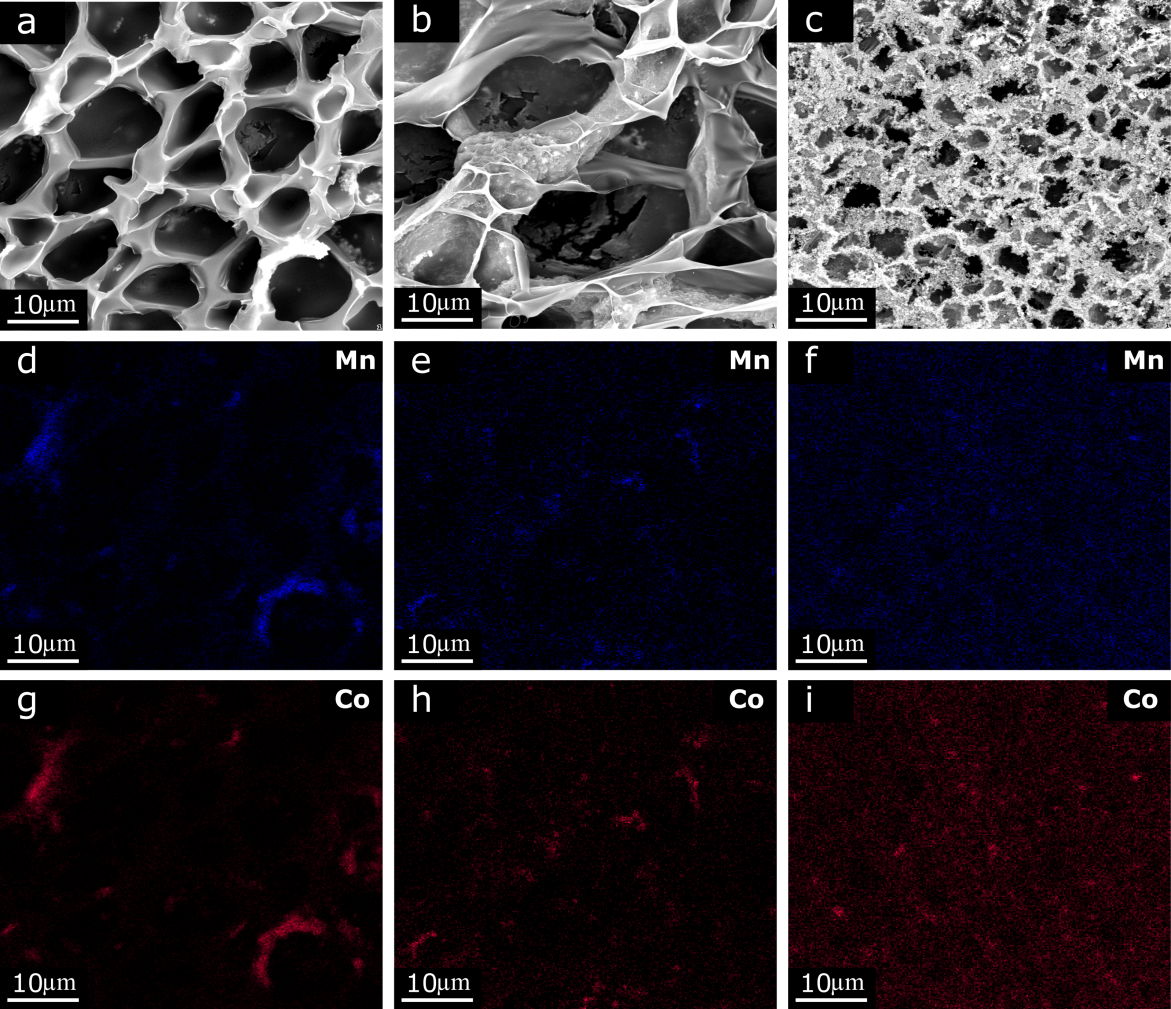
EDS analysis of hydrogel composites: Hgel-MCO (a, d, g), Hgel-MCO-cCB 1:3 (b, e, h), Hgel-MCO-cCB 1:6 (c, f, i).

Elemental mapping by energy-dispersive X-ray spectroscopy (EDS) confirmed the presence of MCO particles inside the hydrogel structure (Figure 2 and Supporting Information File 1, Figure S3). It was not possible to detect the presence of conductive carbon (cCB) particles due to limitations of the instruments and the presence of carbon atoms also in the polymer chains forming the hydrogel skeleton. As mentioned above, the higher the concentration of cCB, the more MCO catalyst particles were found outside the surface of the polymer scaffold. In addition, it can be concluded that in the case of a hydrogel sample with Hgel-MCO catalyst particles (Figure 2a,d, and g), the dispersion of these particles is quite poor and MCO aggregates were visible. With the increase in the concentration of particles added to the hydrogel, the number of visible aggregates of MCO particles decreases (Figurs 2b,c,e,f,h, and i). In the case of a sample where the concentration of particles of conductive carbon reached the value of 54.6 mg/mL (Hgel-MCO-cCB 1:6), much smaller MCO aggregates were visible (Figure 2c,f, and i). The average diameter of the used commercial MnCo_2_O_4_ particles after 0.5 h in an ultrasound bath was 204.5 ± 92.9 nm [46]. Also, their arrangement seems to be much more homogeneous than that in the case of composites with a lower concentration of cCB particles. The dispersion of the catalyst particles in the hydrogel helps to avoid or significantly reduce the formation of aggregates, which may result in an increase of the active surface area of the catalyst, and thus affect its efficiency [20]. Therefore, future research should include the development of a better method of dispersing MCO and cCB particles in the hydrogel precursor solution to obtain a more homogeneous distribution of these particles in the structure of the polymer matrix and on its surface.

**Figure 3:**
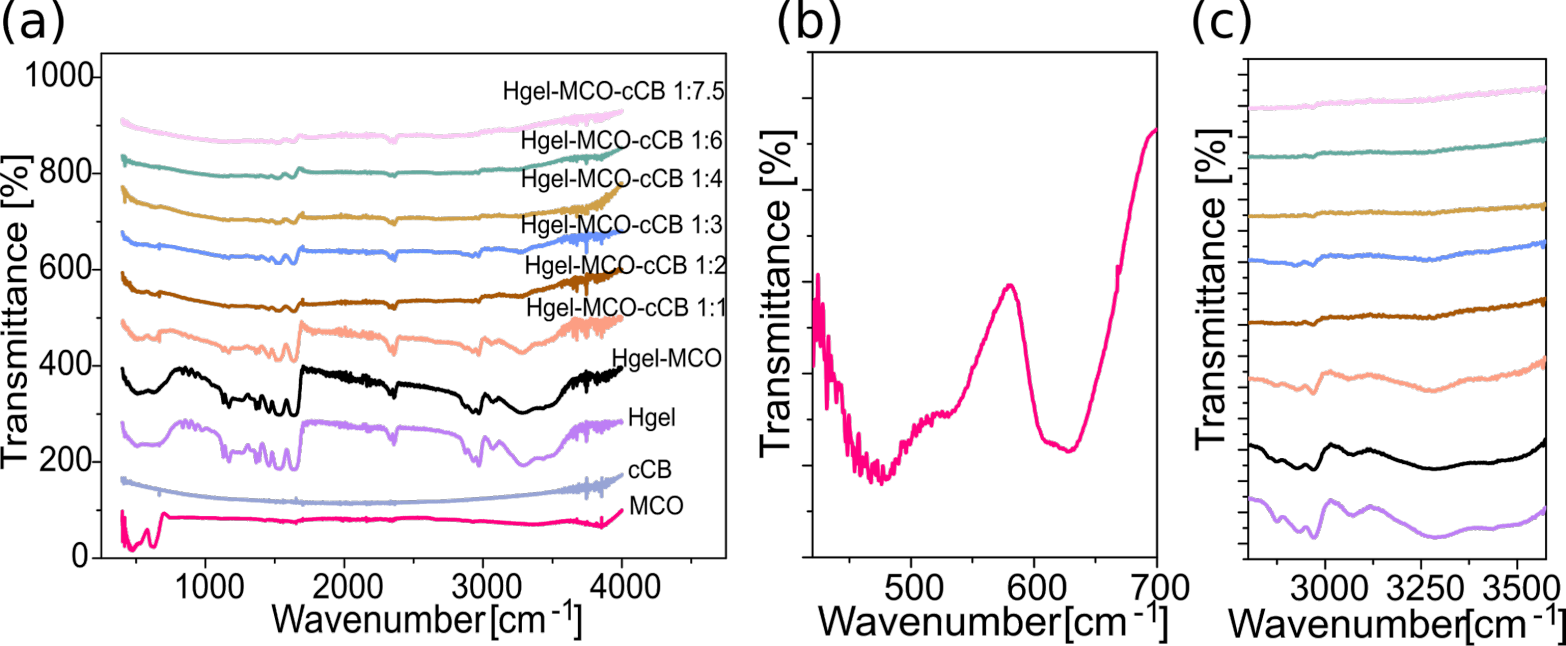
Transmittance plot in the function of wavenumber based on absorbance data from FTIR-ATR spectra of hydrogel composites and their components (a). FTIR graph of MnCo_2_O_4_ strongest characteristic transmittance peaks (b). Comparison of the intensity of P(NIPAAm) strongest characteristic transmittance peaks with different conductive carbon black concentrations in hydrogel composite samples (c).

The Fourier-transform infrared spectroscopy (FTIR) results of the hydrogel compositions and their pure components are shown in Figure 3a. The spectra present the strongest characteristic transmittance peaks of PNIPAAm at 3430, 3284, and 3068 cm^−1^, which are assigned to the N−H stretching of the secondary amine. Peaks at 1637 and 1535 cm^−1^ can be ascribed to the stretching of the carbonyl group C=O [42,48]. In the FTIR spectrum of MnCo_2_O_4_, two transmittance bands at 620 and 478 cm^−1^, characteristic of M–O stretching and vibrations of the spinel structure, were visible (Figure 3b) [49–50]. In the case of super P Li conductive carbon black, no distinct peaks can be seen in the FTIR spectra. However, it is visible that with the increase in the amount of cCB in the structure of the hydrogel, the characteristic peaks of the aforementioned functional groups forming the hydrogel become less and less intense (Figure 3c). It can therefore be assumed that the high concentration of conductive carbon particles overrides (blocks) signals from other compounds.

### Electrical properties of hydrogel composites

Electrochemical impedance spectroscopy was employed to characterise the electrical properties of hydrogel-based structures. The impedance modulus |Z| decreased with the increase in the amount of cCB contained in the hydrogel structure. The changes are within the range of 79.7–1390.5 kΩ at 0.01 Hz and 3.5–18.6 kΩ at 1 Hz.

**Figure 4:**
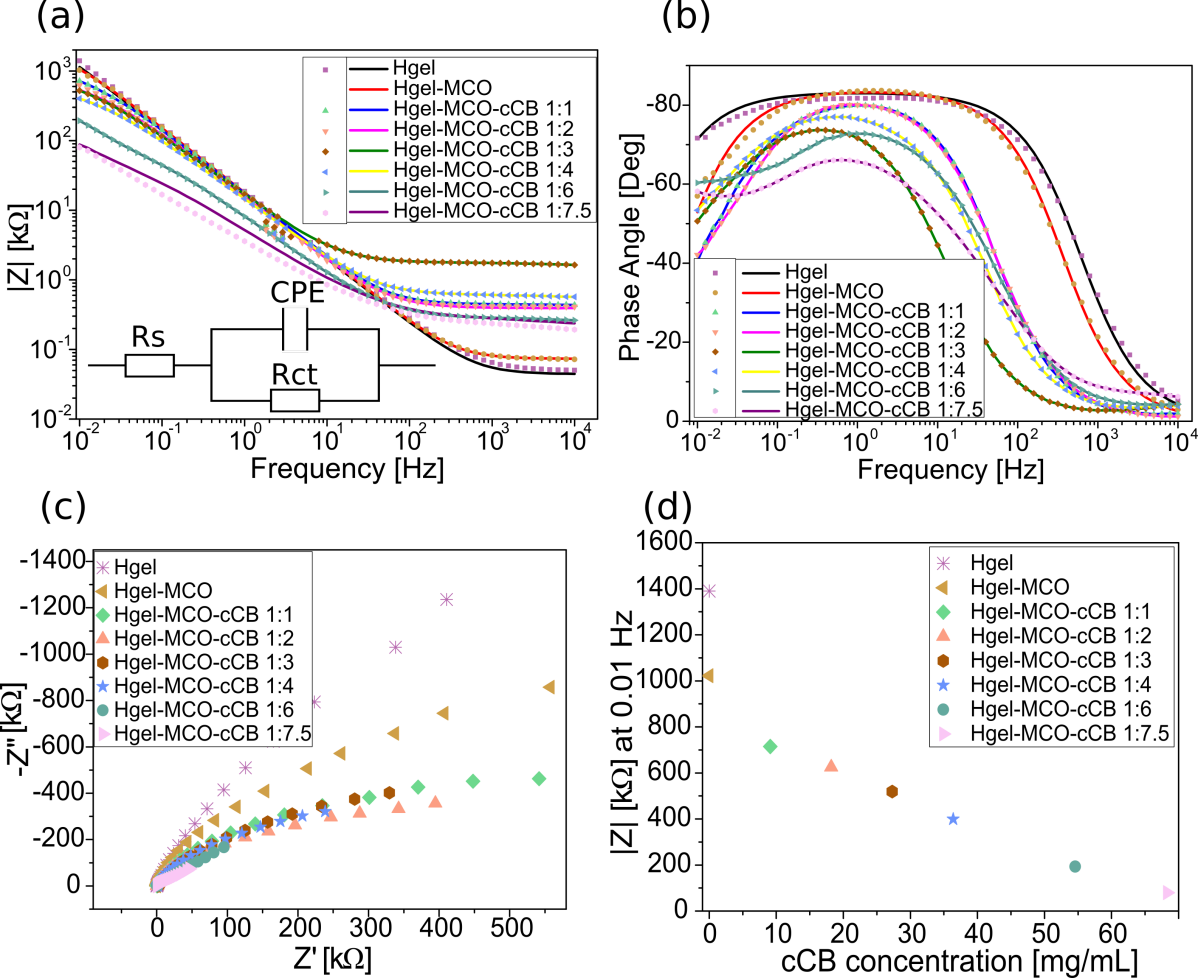
Analysis of electrical properties of hydrogel-based samples with catalyst (M CO) and conductive (cCB) particles (experimental data (dot line) and Randles model results (solid line)): a) Bode modulus and b) phase angle with fitting curves, and c) Nyquist plots of hydrogel samples. d) Dependence of the hydrogel |Z| on the cCB concentration at 0.01 Hz.

The trend of the electrochemical impedance spectroscopy (EIS) results shows the capacitive-dominant nature of the hydrogel for all analysed hydrogel-based electrodes. Three different regions were observed in the Bode plots for the hydrogel samples tested. At the highest frequencies (104–103 Hz and 104–102 Hz for samples without and with cCB, respectively), a resistive behaviour is observed (Figure 4a), while the phase shift is close to 0° (Figure 4b). For hydrogels containing MCO-cCB, the capacitive behaviour starts at 200 Hz, while for samples without cCB, from 2000 Hz. Depending on the sample, the phase angle reaches a maximum of −90° to −65°. The Nyquist plot (Figure 4c) presented similar profiles for all the samples. In the case of samples without cCB and with cCB content not higher than 36.4 mg/mL, initiation of a semicircular area was observed at higher frequencies, which did not happen at lower frequencies. The two hydrogel samples with the highest cCB contents showed the lowest impedance, as indicated by the smallest diameter of the semicircles [51]. It is visible that the electrical properties strongly depend on the conductive carbon concentration (Figure 4d). A similar EIS behaviour was observed in the literature for several different hydrogel-based structures [26,52–54]. For comparison, the impedance values of gelatin methacryloyl (GelMA), GO/GelMA, and r(GO/GelMA) at 1 Hz were 193, 98, and 10 kΩ, respectively [14]. Wu et al. obtained an impedance magnitude in the range of 3–7 kΩ at 1 Hz for PEGda-PANI hydrogels [51]. Comparable values were obtained for PTAA/MAAG-based homogeneous electronically conductive double-network hydrogels [53]. These hydrogel materials were tested for biomedical applications. Nevertheless, they show that the impedance trend that we obtained is similar to that of other hydrogel materials presented in the literature. Additionally, experimental EIS data was modelled with simple electrical equivalent circuit (insert in Figure 4a), where *R*_s_ corresponds to solution resistance, *R*_ct_ is related to charge transfer resistance at the interface of the solution and the electrode material, while CPE is a constant phase element, which represents capacitive performance of the electrode. The circuit parameters and their standard deviations were determined for each sample (Table 1).

**Table 1:** The circuit parameters with corresponding standard deviations for the studied samples.

Sample	*R*_s_/Ω	Q*/µF∙s^(α−1)^	α/–	*R*_ct_/kΩ

Hgel	44.4 (1.1%)	10.8 (0.6%)	0.9 (0.1%)	5 500.0 (10%)
Hgel-MCO	73.2 (0.6%)	10.1 (0.4%)	0.9 (0.1%)	2 000.0 (2.3%)
Hgel-MCO-cCB 1:1	440.3 (0.3%)	10.7 (0.3%)	0.9 (0.1%)	983.3 (1.1%)
Hgel-MCO-cCB 1:2	494.1 (0.6%)	12.4 (0.6%)	0.9 (0.2%)	737.2 (1.8%)
Hgel-MCO-cCB 1:3	1712 (0.6%)	13.0 (0.6%)	0.8 (0.3%)	1 100.0 (3.4%)
Hgel-MCO-cCB 1:4	583.4 (1.0%)	15.2 (1.0%)	0.8 (0.4%)	980.7 (5.3%)
Hgel-MCO-cCB 1:6	264.9 (1.5%)	32.1 (1.4%)	0.8 (0.6%)	574.0 (9.5%)
Hgel-MCO-cCB 1:7.5	250.0 (1.5%)	57.4 (1.3%)	0.7 (0.6%)	426.1 (9.5%)

*Impedance (*Z*) of CPE: *Z*_CPE_ = 1/*Q*(jω)^α^, where *Q* corresponds to the capacitance.

The relative standard deviations for all parameters are in the range between 0.1% and 9.5%. The capacitive parameter (*Q*) is 10.8 µF∙s^(α−1)^ for pure hydrogel, and after the addition of MCO and cCB 1:1 *Q* slightly decreases. It gradually increases with a higher content of cCB in the hydrogel-MCO structure. For the studied materials, CPE can be linked to a surface distribution of properties, related to the material roughness and the porosity of the films. In general, α values correlate with the observed behaviour of the corresponding *Q* parameters (Table 1). Therefore, the results indicate that microporosity, and thus the electroactive surface area of the electrode, increases with a higher content of cCB in the hydrogel-MCO film. A slightly different trend can be observed for the charge transfer resistance *R*_ct_ at the solution–film interface. The highest *R*_ct_ is observed for pure hydrogel (5500.0 kΩ), which is in agreement with previous results. After addition of MCO and cCB 1:1 there is a significant decrease in *R*_ct_ down to 2000 kΩ, indicating improved electrical properties of the electrode. Then, *R*_ct_ decreases with a higher content of cCB up to 1:2. This sudden increase in *R*_ct_ can be observed for hydrogel-MCO with cCB 1:3. Then the resistance decreases down to 426.1 kΩ for Hgel-MCO-cCB 1:7.5. The most probable reason of this sudden *R*_ct_ increase can be related to an unfavourable ratio of MCO to cCB suspended in the hydrogel matrix for the reaction at the solution–electrode interface. The latter can be also confirmed by the relatively high value of the solution resistance (1712 Ω) compared to that of the rest of the studied samples.

### Application of the hydrogel-based composite in oxygen evolution reaction for water splitting

**Figure 5:**
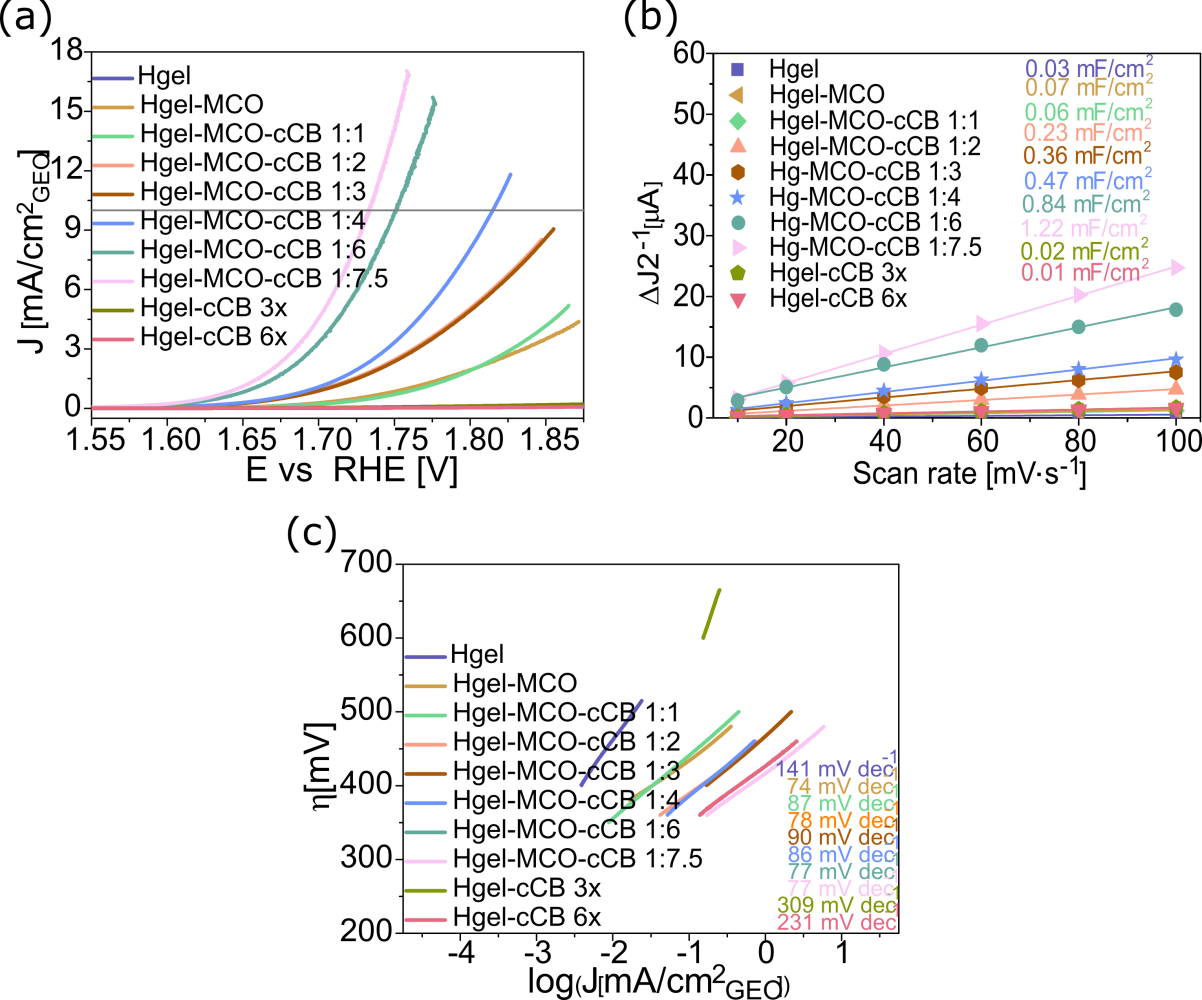
Catalytic activity in the OER process of MCO particles dispersed in the hydrogel: a) polarisation curves, b) double-layer capacitance (*C*_dl_), c) Tafel plots.

The improvement in the electrical properties resulting from an increase in conductive carbon particles is also visible in the OER catalytic activity (Figure 5). The linear sweep voltammetry polarisation curves (Figure 5a) showed that an increase in the cCB contribution to the hydrogel matrix translated into an increase in the number of conduction paths, as manifested by a higher yield of catalyst particles. The potential required to drive a 10 mA/cm^2^ OER current density using the hydrogel with a higher amount of conductive particles compared to catalyst particles (MCO/cCB equal 1:7.5) is 1.73 V versus the reversible hydrogen electrode (RHE), which means that the overpotential was 500 mV. Reducing the conducting carbon (MCO/cCB 1:4) by almost half increased the potential to 1.81 V, and thus the overpotential to 630 mV. For samples with a smaller amount of cCB, pure hydrogel, and hydrogel with MCO catalyst, it was impossible to determine the overpotentials at the standard current density value of 10 mA/cm^2^. Also, hydrogel samples with conductive carbon particles in their three-dimensional structure (Hgel-cCB 3x, Hgel-cCB 6x) and without MCO catalyst particles, showed almost flat polarization curves, which made it impossible to determine the overpotential value at 10 mA cm^−2^ (Figure 5a). These results show that an increase in cCB concentration in the absence of the MCO catalyst is manifested by the lack of any catalytic activity of electrode in the OER process. For comparison, the OER activity of the PPy/FeTCPP (polypyrrole/(4-carboxyphenyl)porphyrin hydrogel) and PPy/FeTCPP/Co catalyst at 10 mA/cm^2^ current density, and in 0.1 M KOH, were 1.74 and 1.61 versus RHE, respectively (the catalyst loading equals to 0.3 mg/cm^2^) [55]. The analysis of 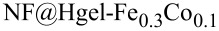, 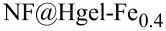, and 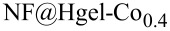 hydrogel catalysts in 1 M KOH for the OER process showed that the overpotential at a current density of 20 mA/cm^2^ was 280 mV, 320 mV, and 370 mV, which corresponds to 1.51 V, 1.55 V, and 1.60 V vs RHE, respectively [56]. However, they used more than four times the loading of the catalyst (1 mg/cm^2^) than we did (0.23 mg/cm^2^). Chen et al. obtained overpotential values of 400 mV (corresponds to 1.6 V vs RHE) at 145.3 mA/cm^2^ for N-doped graphene hydrogels/NiCo in 0.1 M KOH [33].

The electrode double-layer capacitance (*C*_dl_) increased with the increase in cCB particle concentration (Figure 5b). For the pure hydrogel electrode, the double-layer capacitance was equal to 0.03 mF/cm^2^. The addition of cCB at a proportion of 1:1 with MCO caused the *C*dl value to double. The hydrogel electrode, Hgel-MCO-cCB 1:7.5, increased the double-layer capacitance to 1.22 mF/cm^2^, and thus increased it over 20 times compared to that of the Hgel-MCO-cCB 1:1 sample. Tang et al. obtained *C*_dl_ values of 7.91 mF/cm^2^, 9.26 mF/cm^2^, and 4.38 mF/cm^2^, respectively, for 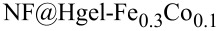, 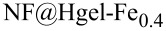, and 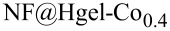 electrodes [56], but with four times higher catalyst loading.

The catalytic kinetics of the hydrogel samples showed a much lower Tafel slope value for pure hydrogel (141 mV/dec) than those obtained for the hydrogel with catalyst and conductive particles dispersed inside the structure of the hydrogel (75–90 mV/dec) (Figure 5c). Depending on the potential applied and the process condition, the water oxidation could be a one to four electron transfer process [57–58]. Tafel slope values in the range of 75–90 mV·dec^−1^ represent a possible mixed mechanism (two or three electron transfer processes), with a strong influence of two electron transfers. Comparing the value of the Tafel slope of the hydrogel composite containing MCO and cCB particles with the Tafel slope of pure hydrogel (141 mV·dec^−1^), it is visible that the addition of the catalyst significantly accelerated the kinetics of the reaction. The increase in the amount of conductive carbon in the structure of the hydrogel composite does not affect the further acceleration of the kinetics, although an increase in the catalytic activity is visible, represented by a decrease in the overpotential (Figure 5a). This is related to the increase of the electrode double-layer capacitance with the increase in the amount of cCB (Figure 5b). This confirms the comparable rate and mechanism of the OER reaction of the Hgel-MCO-cCB electrode. Comparing our Tafel slope with the values obtained for the PPy/FeTCPP and PPy/FeTCPP/Co samples (74 mV/dec and 65 mV/dec, respectively), they are very similar [55], and much better than the results obtained for NF-NiCo (614 mV/dec) [34]. For transition-metal-functionalised polyaniline-phytic acid hydrogels, Tang et al. obtained Tafel slopes in the range of 78–191 mV/dec [56].

The results obtained confirm that ensuring the appropriate concentration of ion-conducting and electron-conducting particles in the hydrogel structure is a necessary condition to provide high electrocatalytic efficiency of the catalyst particles participating in the oxygen evolution reaction. Table 2 compares the results obtained for the hydrogel samples investigated by us with the data of hydrogel-based electrodes obtained by other scientific groups. Comparing the overpotential values obtained by us and data obtained by other groups for hydrogel electrodes with various types of electrocatalysts, there is no improvement in the catalytic efficiency due to the procedure we used to produce the catalytic layer. However, the important parameter here is the "loading of the catalyst", which tells about the amount of catalyst used to prepare the hydrogel-based electrode. It is visible that the data of other research groups compared in Table 2 concerned samples in which the catalyst content (expressed as the loading of catalyst) was higher, and in the case of studies where the overpotential values were even twice as high as those obtained by us, even over four times higher [56]. The results presented in this work indicate a promising direction for further work aimed at obtaining electrocatalytic layers participating in the OER process and characterized by high efficiency, while using a simple one-step method for synthesizing a conductive hydrogel containing electrochemically active particles.

**Table 2:** Comparison of results of OER performed with the hydrogel-based electrode reported in this work and in the literature.

Sample	Catalyst	η [mV]	η at 25 µA·cm^−2^_ox_ [mV]	double-layer capacitance [mF]	Tafel slope [mV·dec^-1^]	Loading of catalyst [mg·cm^−2^]	Ref.

Hgel	–	–^a,c^	–	0.03	141	–	this work
Hgel-MCO	MCO	–^a,c^	486^a,f^	0.07	74	0.23	this work
Hgel-MCO-cCB 1:1	MCO	–^a,c^	496^a,f^	0.06	87	0.23	this work
Hgel-MCO-cCB 1:2	MCO	–^a,c^	432^a,f^	0.23	78	0.23	this work
Hgel-MCO-cCB 1:3	MCO	–^a,c^	436^a,f^	0.36	90	0.23	this work
Hgel-MCO-cCB 1:4	MCO	630^a,c^	437^a,f^	0.47	86	0.23	this work
Hgel-MCO-cCB 1:6	MCO	520^a,c^	397^a,f^	0.84	77	0.23	this work
Hgel-MCO-cCB 1:7.5	MCO	500^a,c^	388^a,f^	1.22	77	0.23	this work
Hgel-cCB 3x	–	–^a,c^	–	0.02	309	–	this work
Hgel-cCB 6x	–	–^a,c^	–	0.01	231	–	this work
PPy/FeTCPP	Fe	510^a,c^	–	–	74	0.3	[55]
PPy/FeTCPP/Co	Fe, Co	380^a,c^	–	–	65	0.3	[55]
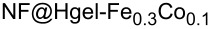	Fe_0.3_Co_0.1_	280^b,d^	–	7.91	78	1	[56]
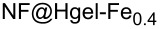	Fe_0.4_	320^b,d^	–	9.26	96	1	[56]
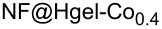	Co_0.4_	370^b,d^	–	4.38	87	1	[56]
N-doped graphene hydrogels/NiCo	NiCo	400^a,e^	–	–	614	–	[33]
NiO-CS	NiO	440^b,c^	–	–	38	0.6	[34]
[Ni,Fe]O-CS Ni/Fe 2:1	[Ni,Fe]O	317^b,c^	–	–	32	0.6	[34]

^a^Analyses were performed in 0.1 M KOH; ^b^analyses were performed in 1 M KOH; ^c^η at 10 mA/cm^2^; ^d^η at 20 mA/cm^2^; ^e^η at 145.3 mA/cm^2^; ^f^catalyst active surface area equal 3.195 cm^2^ (for 45 µg of MCO per electrode).

## Conclusion

The article presents a one-stage method for the fabrication of polymer composites based on a hydrogel in which MnCo_2_O_4_ as the catalyst (MCO) and conductive carbon black particles as conductive fillers have been embedded. In the developed synthesis method, the need to use high temperature was omitted, which is an element that distinguishes the applied procedure from others described in most literature reports. Scanning electron microscopy studies confirmed the fabrication of a composite polymer containing MCO catalyst particles and conductive carbon cCB particles either caught inside or bound to the surface of the hydrogel. Increasing the concentration of conductive carbon particles to a value that prevented their retention in the hydrogel matrix caused MCO and cCB particles to also be present on the surface. After the freeze-drying process, the hydrogel composites were characterised by their porosity, as the pores filled with water in the hydrated state become empty after water sublimation. With the increasing concentration of cCB, the size and number of pores decreased. In the process of electrocatalysis, it is crucial that the size and most importantly, the number of pores, are as large as possible. The porous microstructure of the hydrogel matrix is capable of swelling and thus accommodating significant amounts of ionic liquids. Such a structure provides constant access of the electrolyte molecules/ions to the catalyst particles surrounding it, increasing the speed and efficiency of the electrochemical reaction. Thereby, efficient electrocatalytic processes are promoted. By measuring impedance, we demonstrated the dependency of the hydrogel electrical properties on the concentration of conductive particles suspended in the three-dimensional structure. In addition, the possibility of using the produced hydrogel composites as electrocatalytic electrodes for the oxygen evolution reaction was presented. The results showed that, as predicted, the higher the concentration of conductive particles, the higher the electrocatalytic efficiency of the MCO particles. The separation of catalyst particles suspended in the hydrogel and their homogeneous dispersion, combined with the presence of an optimised concentration of conductive particles, responsible for the transport of ions and electrons in the hydrogel structure, favour the increase in the efficiency of the OER process. Further work on these types of three-dimensional materials seems to be a promising direction in the search for new active electrodes participating in the water-splitting reaction. The realisation of nanostructured materials plays a key role in the development of the desired materials. Adopting an appropriate strategy for designing and producing hydrogel composites and modifying their molecular architecture will allow them to meet critical challenges in advanced energy technologies. Ultimately these hydrogels will overcome the limitations of current materials, improving the efficiency of devices in the field of storage and conversion of energy.

## Experimental

### Materials

*N*-isopropylacrylamide (NIPAAm, 97%, Sigma-Aldrich, Poland), *N*,*N*′-methylenebisacrylamide (BIS-AAm, 99.5%, Sigma-Aldrich, Poland), 2-hydroxy-4′-(2-hydroxyethoxy)-2-methylpropiophenone (Irgacure 2959, 98%, Sigma-Aldrich, Poland), ammonium persulfate (APS, 98%, Sigma-Aldrich, Poland), *N*,*N*,*N*′,*N*′-tetramethylethylenediamine (TEMED, 99%, Sigma-Aldrich, Poland), commercially available manganese cobalt spinel MnCo_2_O_4_ powder (MCO, Marion Technologie, France), Super P Li conductive carbon black (cCB, Imerys, Belgium), 0.1 M KOH (Titripur^®^, Merck, Germany).

### Hydrogel sample preparation

**Figure 6:**
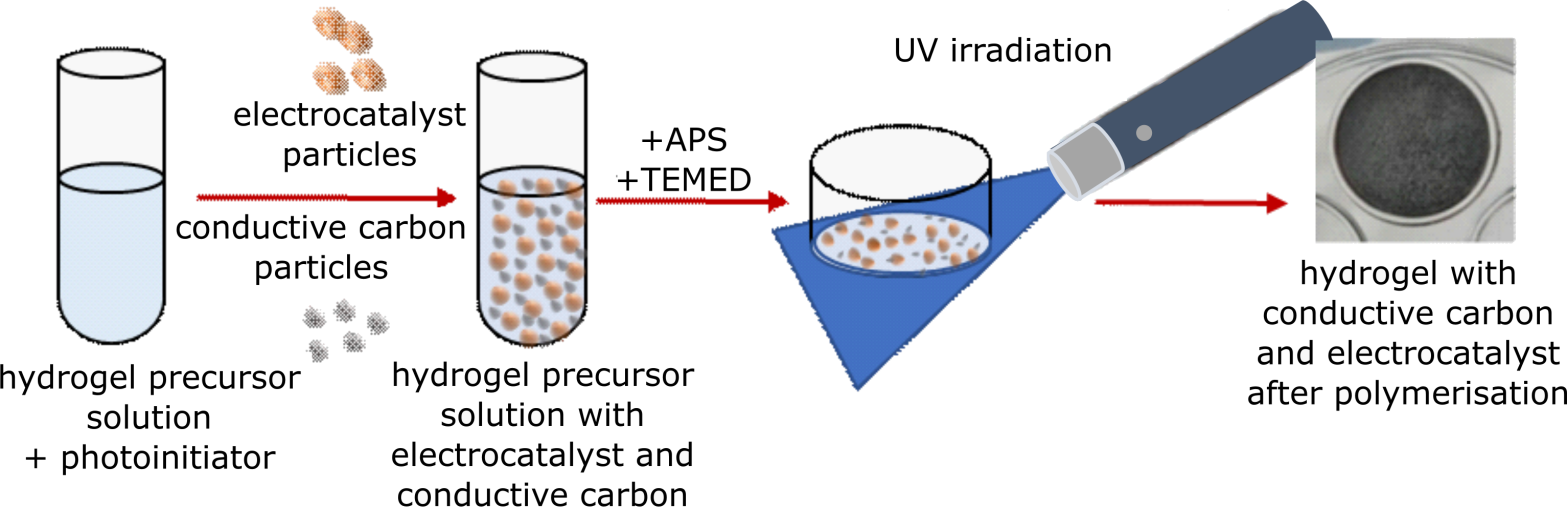
Scheme of hydrogel polymerisation process with electrocatalyst and conductive carbon particles.

Hydrogels with conductive and catalyst particles dispersed throughout were synthesised via a one-step polymerisation procedure – all components were dissolved or suspended in a hydrogel precursor solution (Figure 6). The hydrogel precursor solution was a composition of NIPAAm, used as the main monomer, and BIS-AAm as a cross-linker in the proportion 35:1, dissolved in deionised water (90 wt %). The water solution contained MnCo_2_O_4_ as electrocatalyst particles and conductive carbon black particles as carbon-based conductive fillers. Two types of initiating agents were used in the hydrogel polymerisation process: 2-hydroxy-4′-(2-hydroxyethoxy)-2-methylpropiophenone (as a photoinitiator used to trigger the hydrogel polymerisation reaction upon UV irradiation) as well as APS and TEMED. The addition of APS and TEMED was necessary as there is a need to quickly increase the viscosity of the hydrogel precursor solution to avoid the sedimentation of suspended particles (MCO and cCB). Table 3 presents the amounts of cCB used in the preparation of each conductive hydrogel sample. Ultraviolet irradiation (UV EMITA VP-60, 180 W, 220–240 V AC) in an ice bath lasted 2–4 minutes for complete polymerisation.

**Table 3:** The amounts of conductive carbon added to the hydrogel precursor solution and the UV irradiation time used to polymerize individual samples. The hydrogel precursor solution had a constant composition regardless of the sample: NIPAAm = 97.2 mg/mL; BIS-Aam = 2.8 mg/mL; APS = 75 µL/mL; TEMED = 7.5 µL/mL; Irgacure = 5 mg/mL; as well as a constant concentration of the catalyst MCO = 9.1 mg/mL.

Sample		cCB		UV [minute]

1	Hgel-MCO	–		2
2	Hgel-MCO-cCB 1:1	9.1 mg/mL	5.7 vol %	2
3	Hgel-MCO-cCB 1:2	18.2 mg/mL	11.3 vol %	2
4	Hgel-MCO-cCB 1:3	27.3 mg/mL	17.1 vol %	3
5	Hgel-MCO-cCB 1:4	36.4 mg/mL	22.8 vol %	3
6	Hgel-MCO-cCB 1:6	54.6 mg/mL	34.2 vol %	4
7	Hgel-MCO-cCB 1:7.5	68.3 mg/mL	42.8 vol %	4
8	Hgel-cCB 3x	27.3 mg/mL	17.1 vol %	3
9	Hgel-cCB 6x	54.6 mg/mL	34.2 vol %	4

In contrast to numerous works in which elevated temperatures were used for the synthesis of hydrogel composites, here the process of mixing the hydrogel precursor solution with MCO and cCB was performed in a cooling bath. Similarly, the polymerisation process was performed using a cooling bath to eliminate the high temperature generated by UV irradiation. In both cases, we protected the PNIPAAm polymer from reaching the lower critical solution temperature (LCST) by cooling. The LCST of PNIPAM in pure water is approx. 32 °C [59–61]. Polymerisation reactions performed at a temperature above LCST results in shrinkage of the hydrogel structure and formation of inhomogeneities. Such a hydrogel ceases to be transparent and loses most of its water [42].

### Morphological and physicochemical characterisation

Scanning electron microscopy was performed with an FE-SEM FEI Quanta FEG 250 at an accelerating voltage of 10 kV. Before imaging, the hydrogel samples were freeze-dried and coated with a 10 nm gold layer.

The chemical composition of the hydrogel was analysed with a Thermo Fisher Scientific silicon drift detector energy-dispersive X-ray spectroscope.

For characterisation of the present material functional groups, FTIR spectroscopy in attenuated total reflectance (ATR) mode was used (Bruker Vertex70 FT-IR Spectrometer). The FTIR analysis was carried out in a wavenumber range of 400–4000 cm^−1^ and with a resolution of 2 cm^−1^ and eight scans were made for each sample. Transmittance data was calculated based on absorbance. Before analysis, all hydrogel samples (pure and with MCO and cCB) were freeze-dried. The raw data were manipulated by baseline correction, normalised, and smoothed.

### Measurements of electrical properties

**Figure 7:**
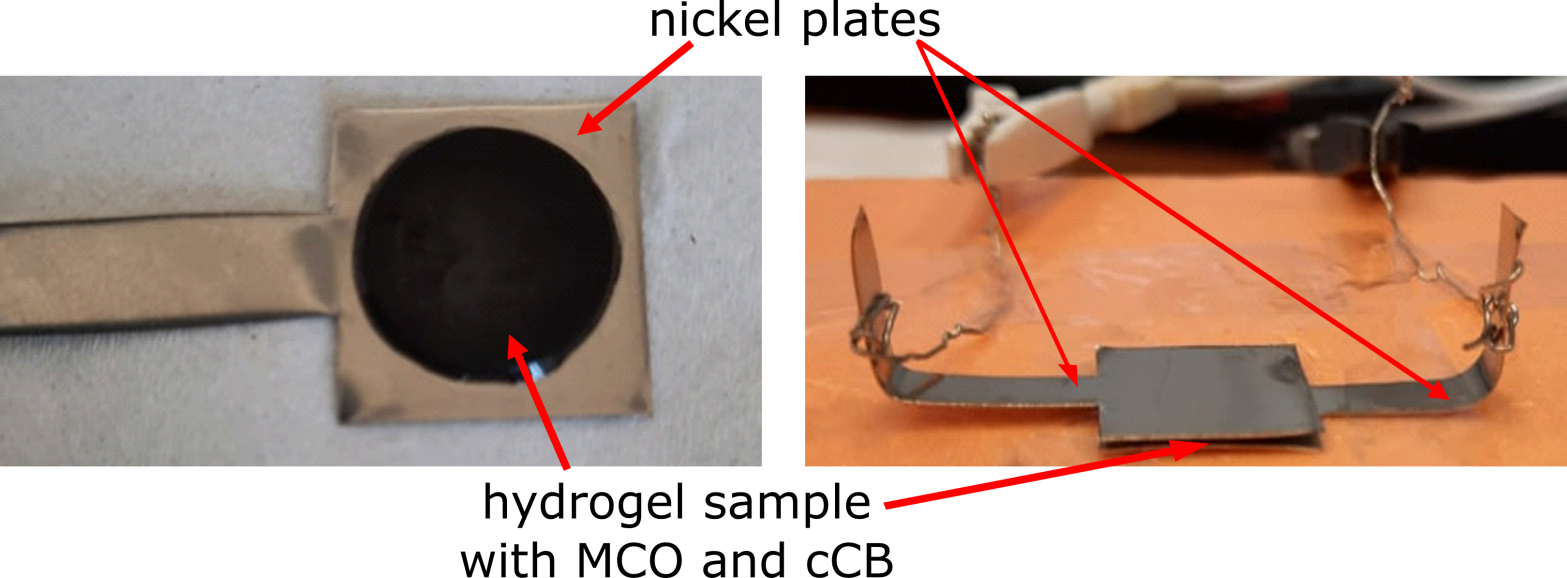
System for measuring electrical properties of hydrogel-based samples with catalyst (MCO) and conductive carbon black (cCB) particles.

The electrical properties of the hydrogels were determined based on EIS measurements using a VersaSTAT 4 potentiostat. For the measurements, a hydrogel in the form of a disc was placed between two nickel plates (2 × 15 × 15 mm) connected with the impedance analyser (2-probe measurement) (Figure 7). The impedance spectrum was acquired in the frequency range of 0.01 Hz – 10 kHz and at an amplitude of 10 mV. The data was analysed by the ZView software.

### Electrode preparation and electrochemical measurements

For the electrochemical analysis, 5 µL of the hydrogel precursor solution was dropped on a rotating glassy carbon (GC) electrode with a working area of 0.196 cm^2^. The OER experiments were performed in a three-electrode system controlled by a BioLogic BP-300 potentiostat/impedance meter in an O_2_-saturated 0.1 M KOH electrolyte (Titripur^®^, Merc, Germany). The rotating ring disk electrode (RRDE-3A) was used at 1600 rpm; GC and Hg/HgO were the working electrode and the reference electrode, respectively. Linear sweep voltammetry (LSV) data was recorded from 1.1 to 2.0 V vs RHE with a 10 mV/s scan rate. The charge transfer resistance (*R*_ct_) was determined based on EIS measurements. The spectra were obtained in the frequency range from 10 kHz to 0.1 Hz at 1.7 V vs RHE, and with an amplitude of 10 mV. All potential values were converted to the RHE and then iR-corrected. Cycling voltammetry scans were performed at scan rates of 10, 20, 40, 60, 80, and 100 mV·s^−1^ to estimate the double-layer capacitance. The non-faradaic potential region was applied (from 1 V to 1.7 V vs RHE).

## Supporting Information

File 1 Additional figures.
