# Supplementary material for: Influence of conductive carbon and MnCo2O4 on morphological and electrical properties of hydrogels for electrochemical energy conversion
Source: Beilstein J Nanotechnol. 2024 Jan 11;15:57–70. doi: 10.3762/bjnano.15.6 (PMC10790649; doi:10.3762/bjnano.15.6)
Supplement: File 1 — Additional figures. [file Beilstein_J_Nanotechnol-15-57-s001.pdf]

BEILSTEIN JOURNAL OF NANOTECHNOLOGY

Supporting Information

for

Influence of conductive carbon and $MnCo_2O_4$ on morphological and electrical properties of hydrogels for electrochemical energy conversion

Sylwia Pawłowska, Karolina Cysewska, Yasamin Ziai, Jakub Karczewski, Piotr Jasiński and Sebastian Molin

Beilstein J. Nanotechnol. **2024,** *15,* 57–70. doi:10.3762/bjnano.15.6

Additional figures

License and Terms: This is a supporting information file under the terms of the Creative Commons Attribution License (https://creativecommons.org/licenses/by/4.0). Please note that the reuse, redistribution and reproduction in particular requires that the author(s) and source are credited and that individual graphics may be subject to special legal provisions.
The license is subject to the *Beilstein Journal of Nanotechnology* terms and conditions: (https://www.beilstein-journals.org/bjnano/terms)

Characterisation of hydrogel-based polymer composites with dispersed catalytic and conductive particles:

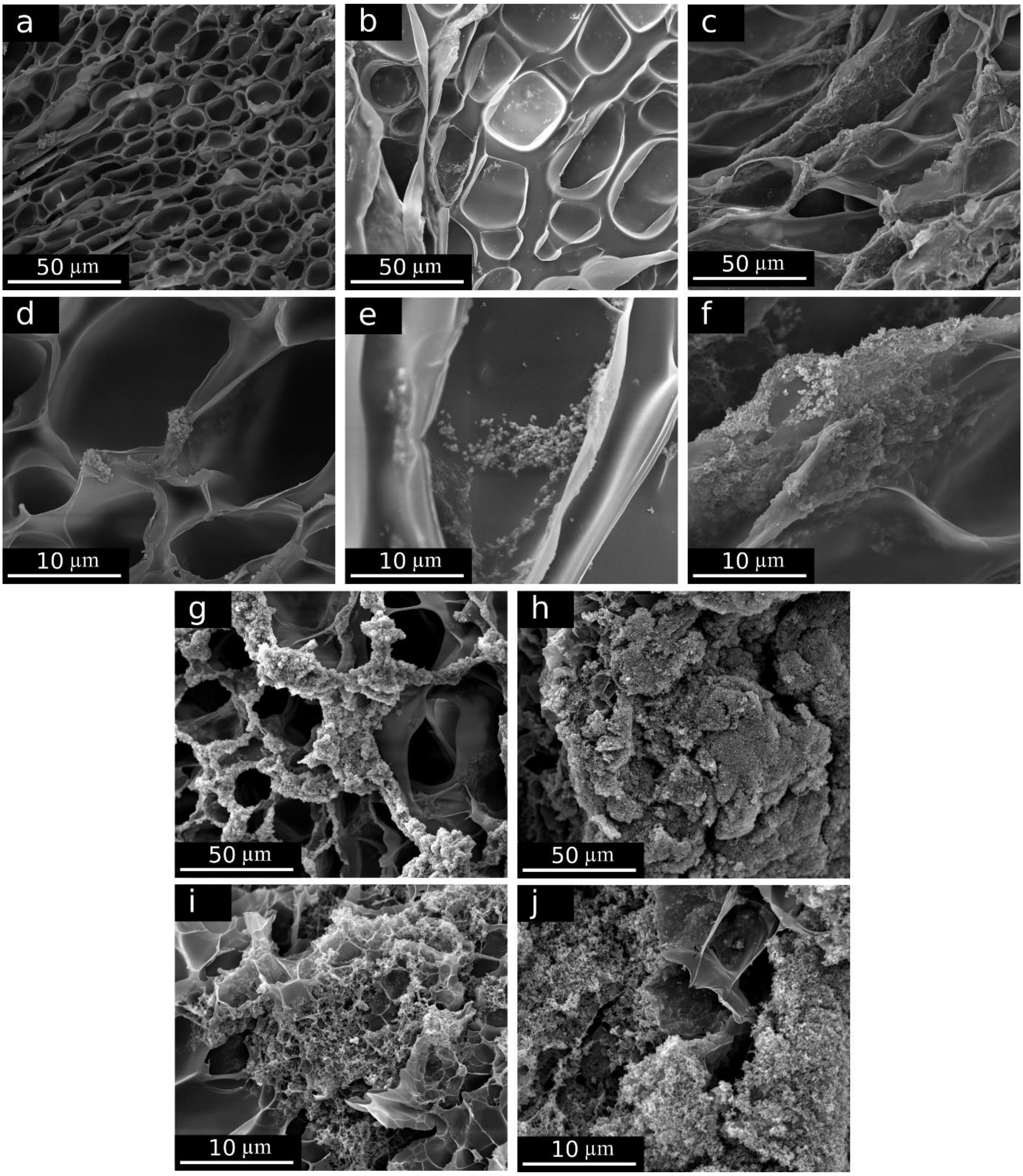

Figure S1: SEM micrographs of freeze-dried nanostructures of composite hydrogel samples: Hgel-MCO (a, d), Hgel-MCO-cCB 1:1 (b, e), Hgel-MCO-cCB 1:2 (c, f), Hgel-MCO-cCB 1:4 (g, i), and Hgel-MCO-cCB 1:7.5 (h, j).

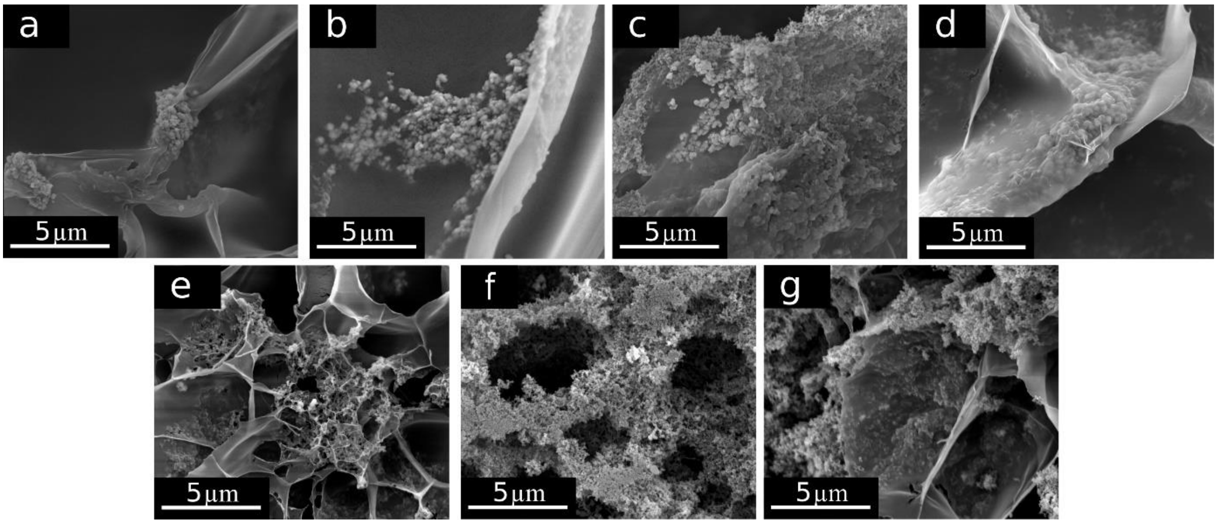

Figure S2: SEM micrographs in higher magnification of freeze-dried nanostructures of composite hydrogel samples: Hgel-MCO (a), Hgel-MCO-cCB 1:1 (b), Hgel-MCO-cCB 1:2 (c), Hgel-MCO-cCB 1:3 (d), Hgel-MCO-cCB 1:4 (e), Hgel-MCO-cCB 1:6 (f), and Hgel-MCO-cCB 1:7.5 (g).

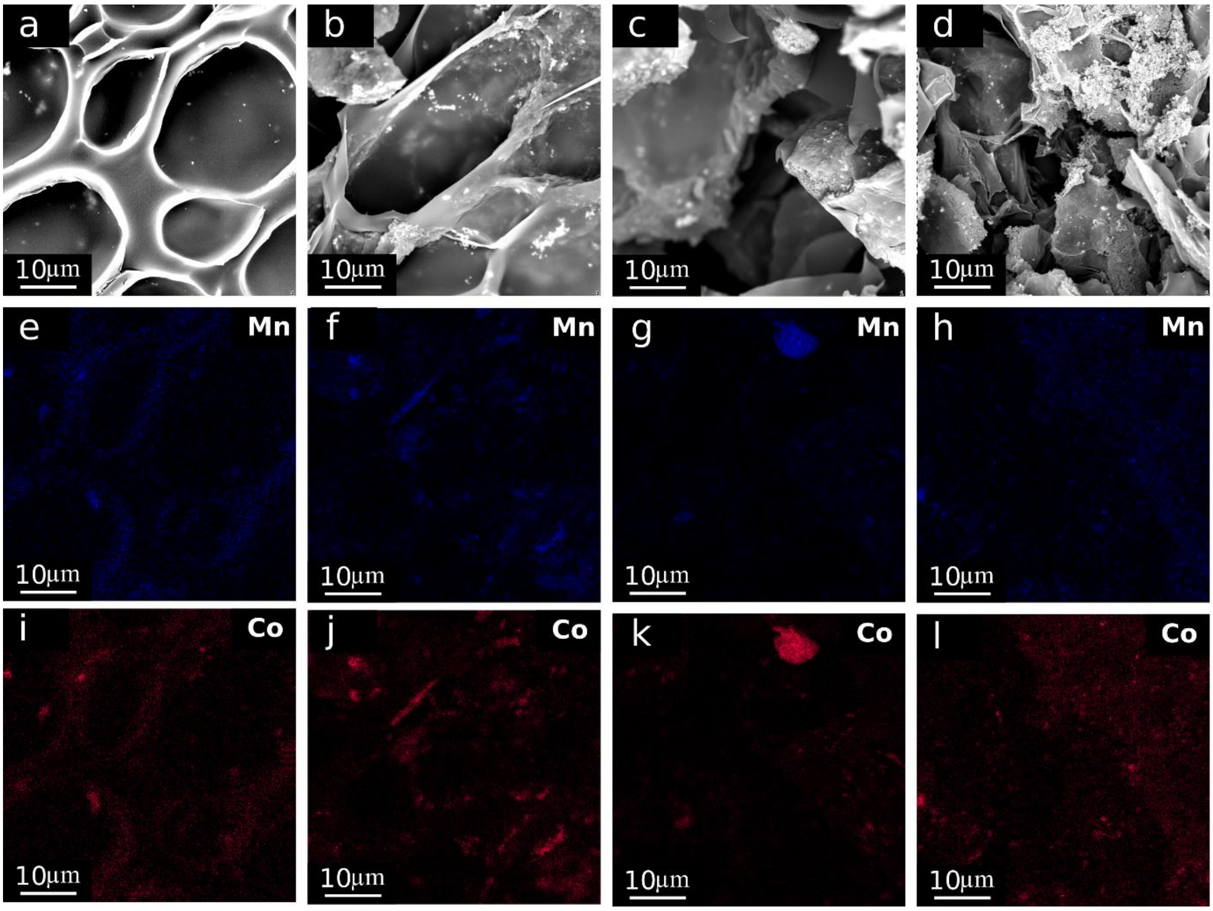

Figure S3: EDS analysis of hydrogel composites: Hgel-MCO-cCB 1:1 (a, e, i), Hgel-MCO-cCB 1:2 (b, f, j), Hgel-MCO-cCB 1:4 (c, g, k), and Hgel-MCO-cCB 1:6 (d, h, l).
